# The Deadly Fly

**Published:** 1907-10

**Authors:** 


### EDITORIAL DEPARTMENT.

### THE DEADLY FLY.

Uncle Toby was evidently not a sanitarian, and had no conception of "germs" or germ carriers. If he had been he would surely have killed that fly. A modern appreciation of the fly recognizes in him and his kinfolks one of the greatest enemies of man, an insidious, indefatigable and most successful disseminator of infectious diseases, and a nuisance generally.

To most people all flies look alike; but there are flies and flies. The little grayish house fly, the most abundant of the species, and known to science as *musca domestica,* has mouth parts separate at the tips for sucking up liquids, but it is not within the power of this species to pierce the cuticle. He gets in his deadly work by crawling over food and distributing the filth (and infection) that adheres to his feet and proboscis when he dines on feces of a typhoid patient, for instance. (Ugh!) It is the stable fly that is thrice armed for death and destruction,—and that is the fellow that comes in from the manure heaps; he can bite, and bite hard, and in addition to the power to scatter infection by crawling over things, like *m. d.,* he can inoculate you, vaccinate you with any number of viruses;—he keeps them on tap, and may, in addition, produce blood poisoning by the introduction of some of the streptococci. He is especially dangerous as a disseminator of the tetanus bacillus. Both he and they breed in manure—the intestine of the horse and mule being the native habitat of the lockjaw bacillus. They are voided in great quantities, and as the manure is allowed to remain on our thoroughfares and dry, they are carried in the dust and go everywhere; and if there is a toy pistol wound or an abrasion from the deadly firecracker anywhere about, he is sure to get into it, and like the inscription in Dante's Inferno—leave hope behind; for about one hundred per cent of such cases of lockjaw die.

Well, the stable fly (which has a harder name than his cousin, to whom we have paid our compliments above, and is known as *stomyx calcitrans*), holds himself in readiness to inject you with a culture of tetanus microbes, night or day, and to aid the dust to the extent of his honest ability in the deadly work. Now, I have said that the house fly can't bite. There are those who will deny this,—and lest Mr. Roosevelt should see this and denounce me as a nature faker, I give mu authorities for the statement: "Studies in Insect Life," Samuel J. Hunter, A. M., Zoology and Entomology, University of Kansas.

The above is by way of introduction to the report to the Austin Mayor and Council by the President of the City Board of Health, Dr. W. J. Mathews, which I present herewith, because what he says applies everywhere as well as to Austin, and these forcible and striking remarks should have wide currency, and our readers, especially board of health fellows, should sit up and take notice.

Dr. Mathews said:

"Referring to an instructive article in the *Statesman* stating that horse manure is a breeding place for house flies, I call your attention to the large horse lot south of the courthouse and jail lot, just across the alley. Many horses are kept there. The filthy manure is drawn out of the stables and left in the middle of the lot to rot in the lot instead of being hauled off. The stench is sometimes terrible, and no other place breeds more flies. The horses belong to deputy sheriffs and policemen. Respectfully, R. HILL."

The owner of the lot stated to me that the statements contained in this postal are exaggerated. I shall assume that the statements are correct as a text for what I have to say. In my remarks on the crusade against the fly nuisance in the issue of last Sunday I proved from the best authorities that the fly as a carrier of contagion is responsible for a large percentage of the cases of typhoid fever which have originated in this city, and that the carefully and scientifically conducted experiments of Dr. L. O. Howard of the Washington Academy of Sciences demonstrated beyond a doubt that 95 per cent of all the flies which infest our cities come from horse manure, their favorite breeding place. The heat generated by such a huge pile of horse manure as that described in the above complaint makes it a splendid incubator for flies. It was also shown in my paper that every adult female fly lays 120 eggs. In these reeking, filthy piles of manure behind our stables the eggs of the fly hatch in eight hours. For a time after being hatched they remain worms, feeding upon horse manure, and in ten days' time they are adult flies, and, securing emigration permits in the shape of wings, they leave their filthy origin in search of food in our homes, often carrying disease and death there. With such prolific breeding in horse manure and in garbage more or less carelessly taken care of, the marvel is that typhoid fever is not more rife than we find it.

The remedy is simple. Bury all horse manure so that flies can not gain access to it, and burn up all manner of garbage. This is the simple solution of the fly problems. It is simple enough, but it is difficult to appeal to people who live in filth. I believe that amid their daily environments their olfactory nerves become functionally defective; in other words, their sense of smell is obtunded. This fact was well illustrated in a recent visit to one of our slaughter houses with a skilled veterinary surgeon. This slaughter house was one of the crudest and filthiest concerns I have ever witnessed. From the roof of this wretched building cobwebs hung suspended at least six inches long, blood was spattered all over the interior of the building. This blood and filth on the walls was the accumulation of years, being at least half an inch thick on the walls. What millions of death-dealing germs infested this building! The meat which we eat daily at our tables comes into contact with these awful surroundings. Within a few feet of this modern abattoir was a hog pen containing about one dozen hogs. These hogs were fed on the entrails of the slaughtered animals.

But often these hogs were unable to consume all the entrails furnished them, and those which were not eaten were left to putrify. Now when you consider these noisome odors common to every hog pen and then add to this the fearful stench of putrifying decomposing entrails, and still further add to this an open privy vault within a few feet of the slaughter house which is frequented daily by a number of negroes, you may form a dim conception of the condition of our slaughter houses without visiting them. But further, millions of flies swarm over these putrefying septic entrails, and after enjoying an appetizing dose of these rotten entrails and human and hog filth combined, they betake themselves to the unscreened slaughter house, where a number of slaughtered animals are suspended from the roof, adorned with bacterial cobwebs, and there assembled by the millions, covering these dead carcasses as with a garment, depositing the vilest filth known to mankind, as well as disease-producing germs, on the meat we consume daily, but not before these unclean guests have appeased their hunger and cleansed their bodies as they revel in the juicy meat.

If any of you think this picture overdrawn, come with me to these places and if you find these facts overstated never believe me again. The veterinary surgeon who visited the slaughter house with me, already described, will confirm my statement that I failed to convince the proprietor of this wretched and disgraceful slaughter house that there was any bad odor about his place. His sense of smell was so blunted and his ideas of sanitary matters so crude and depraved that he seemed completely oblivious of the noisome odors and of his unseemly and unsanitary environments. So it is of the stablemen. He passes the greater part of his life inhaling the noxious odors of the stable and looks as happy and satisfied as if he was inhaling the odor of roses. His olfactories are paralyzed, and we can not wake him up to realize how utterly revolting these unpleasant odors are to normal healthy olfactory nerves and to people who live away from filth and love to breathe God's pure untainted air.

Now, these stable manure heaps must go. They have caused many sad deaths, many untold sorrows, the payment of many large doctor's bills and the expenses of many an unnecessary funeral. Is it not time, then, that we were calling a halt to the barbarous and unsanitary methods pursued by even the best stables in our city in the disposition of their stable manure? Aldermen of the city should wake up to their solemn responsibility of the duties of their office, for in their hands largely depends the destiny of the city of Austin. In their hands, to a certain extent, are the issues of life and death as regards this city. By granting us proper sanitary ordinances many deaths could be averted. There would be fewer heart-broken mothers, there would be fewer funeral marches to the city of the dead. Members of the board of aldermen should join the physicians of Austin and the board of health in their unselfish and philanthropic battle against disease and death. We are fighting strenuously and faithfully to rob death of its victory and promote the health and happiness of every home in our beloved city.

